# Automated contouring and planning pipeline for hippocampal-avoidant whole-brain radiotherapy

**DOI:** 10.1186/s13014-020-01689-y

**Published:** 2020-10-30

**Authors:** Christine H. Feng, Mariel Cornell, Kevin L. Moore, Roshan Karunamuni, Tyler M. Seibert

**Affiliations:** 1 UC San Diego Department of Radiation Medicine and Applied Sciences, Altman Clinical and Translational Research Institute, 9500 Gilman Dr. #0861, La Jolla, CA USA; 2 UC San Diego Department of Bioengineering, La Jolla, CA USA

**Keywords:** Brain metastases, Hippocampal-avoidant whole-brain radiotherapy, Artificial intelligence, Radiotherapy automation, Brain segmentation, Knowledge-based planning

## Abstract

**Background:**

Whole-brain radiotherapy (WBRT) remains an important treatment for over 200,000 cancer patients in the United States annually. Hippocampal-avoidant WBRT (HA-WBRT) reduces neurocognitive toxicity compared to standard WBRT, but HA-WBRT contouring and planning are more complex and time-consuming than standard WBRT. We designed and evaluated a workflow using commercially available artificial intelligence tools for automated hippocampal segmentation and treatment planning to efficiently generate clinically acceptable HA-WBRT radiotherapy plans.

**Methods:**

We retrospectively identified 100 consecutive adult patients treated for brain metastases outside the hippocampal region. Each patient’s T1 post-contrast brain MRI was processed using NeuroQuant, an FDA-approved software that provides segmentations of brain structures in less than 8 min. Automated hippocampal segmentations were reviewed for accuracy, then converted to files compatible with a commercial treatment planning system, where hippocampal avoidance regions and planning target volumes (PTV) were generated. Other organs-at-risk (OARs) were previously contoured per clinical routine. A RapidPlan knowledge-based planning routine was applied for a prescription of 30 Gy in 10 fractions using volumetric modulated arc therapy (VMAT) delivery. Plans were evaluated based on NRG CC001 dose-volume objectives (Brown et al. in J Clin Oncol, 2020).

**Results:**

Of the 100 cases, 99 (99%) had acceptable automated hippocampi segmentations without manual intervention. Knowledge-based planning was applied to all cases; the median processing time was 9 min 59 s (range 6:53–13:31). All plans met per-protocol dose-volume objectives for PTV per the NRG CC001 protocol. For comparison, only 65.5% of plans on NRG CC001 met PTV goals per protocol, with 26.1% within acceptable variation. In this study, 43 plans (43%) met OAR constraints, and the remaining 57 (57%) were within acceptable variation, compared to 42.5% and 48.3% on NRG CC001, respectively. No plans in this study had unacceptable dose to OARs, compared to 0.8% of manually generated plans from NRG CC001. 8.4% of plans from NRG CC001 were not scored or unable to be evaluated.

**Conclusions:**

An automated pipeline harnessing the efficiency of commercially available artificial intelligence tools can generate clinically acceptable VMAT HA-WBRT plans with minimal manual intervention. This process could improve clinical efficiency for a treatment established to improve patient outcomes over standard WBRT.

## Background

Whole-brain radiotherapy (WBRT) remains an important treatment for patients with multiple brain metastases, with over 200,000 cancer patients treated with WBRT in the United States annually [1]. Compared to stereotactic radiosurgery (SRS), WBRT provides better distant intracranial tumor control, at a cost of decreased control of existing intracranial metastases and increased neurocognitive adverse effects [2, 3]. Hippocampal-avoidant WBRT (HA-WBRT) has emerged as an approach to retain the intracranial tumor control of WBRT while minimizing cognitive decline.

Neurocognitive dysfunction following irradiation can occur through depletion of hippocampal neural stem cells as they differentiate to a gliogenic lineage and hippocampal atrophy [4]. Hippocampal dosimetry is associated with long-term decline in list-learning delayed recall [5]. A multi-institutional phase II trial, RTOG 0933, demonstrated that hippocampal-avoidant WBRT (HA-WBRT) provided improved preservation of memory and quality of life compared to historical controls [6]. More recently, NRG CC001, a phase III trial that randomized patients to standard WBRT with memantine or HA-WBRT with memantine, demonstrated better cognitive preservation and quality of life without difference in intracranial tumor control or overall survival [7].

The neurocognitive and quality of life advantages of HA-WBRT over standard WBRT provide a compelling argument for HA-WBRT to be considered the new standard of care for patients with good performance status who will undergo WBRT. However, manual hippocampal contouring and intensity-modulated radiotherapy (IMRT) planning are significantly more complex and time-consuming than the blocks and 3D conformal planning of traditional WBRT [8, 9]. On central review of the OARs used for participants on NRG CC001, only 65.5% of OAR contours were per protocol [7], indicating an unmet need for accurate automated segmentation in the practicing community, despite available contouring atlases. We designed and evaluated a workflow using commercially available artificial intelligence tools for automated hippocampal segmentation and treatment planning to efficiently generate clinically acceptable HA-WBRT plans.

## Methods

### Study design and patients

We retrospectively identified 100 consecutive adult patients who received radiotherapy for brain metastases and had an available brain MRI at UC San Diego between April 2015 and August 2018. Eligible patients had intracranial metastases no closer than 5 mm from the hippocampus. Per clinical routine, all of these brain MRI volumes already had associated contours for the brain and for standard organs-at-risk (OARs), including bilateral lens, bilateral optic nerves, and optic chiasm. This study was reviewed and approved by the UC San Diego Institutional Review Board (IRB #181609).

### Hippocampal segmentation

Thin-slice T1 brain MRIs (full head, native 3D acquisitions) were processed using NeuroQuant (CorTechs Labs, Inc., San Diego, CA, USA), an FDA-approved software that uses three-dimensional T1-weighted MRI datasets to register the patient’s brain to a probabilistic atlas for anatomic labeling. The underlying methodological details have been published elsewhere [10, 11], and an open-source software that uses a similar approach is available for research use [12].Our segmentations were performed as untimed batch processing jobs for convenience. Segmentations of bilateral hippocampi are typically generated in less than 8 min per patient or MRI volume [13, 14]. Outputs from NeuroQuant were converted to RTSTRUCT DICOM files compatible with a commercial treatment planning system, Eclipse version 15.6 (Varian Medical Systems, Palo Alto, CA, USA), using software developed in-house with Matlab (Mathworks, Natick, MA, USA). Automated hippocampal segmentations were reviewed for accuracy by a radiation oncologist using the RTOG 0933 Hippocampal Atlas [9]. Segmentations requiring manual edits were flagged for further investigation but allowed to proceed in the pipeline.

### Knowledge-based planning

The imported segmentations and MRI were automatically registered to the patient’s simulation CT using Eclipse. Hippocampal avoidance regions were generated using 5 mm uniform expansion [7], and planning target volumes (PTV) were generated by subtracting the hippocampal avoidance region from the existing brain contour. A publicly available RapidPlan (Varian Medical Systems, Palo Alto, CA, USA) knowledge-based planning routine [15] was applied for a prescription of 30 Gy in 10 fractions using volumetric modulated arc therapy (VMAT) delivery via four full arcs on a TrueBeam (Varian Medical Systems, Palo Alto, CA, USA) linear accelerator with 120-leaf Millennium multileaf collimators. This RapidPlan routine is available without charge under the General Public License but requires creation of a free user account on Varian’s community platform [15]. Plans were normalized to deliver prescription dose to 95% of the PTV. The processing time, defined as the time from image registration to completion of dose calculation, was recorded using a stopwatch for all cases.

### Plan evaluation

Dose-volume histograms (DVH) were calculated for the PTV and OARs of each automated knowledge-based plan without any manual correction. Results were evaluated based on NRG CC001 dose-volume objectives (Table 2).

## Results

**Fig. 1:**
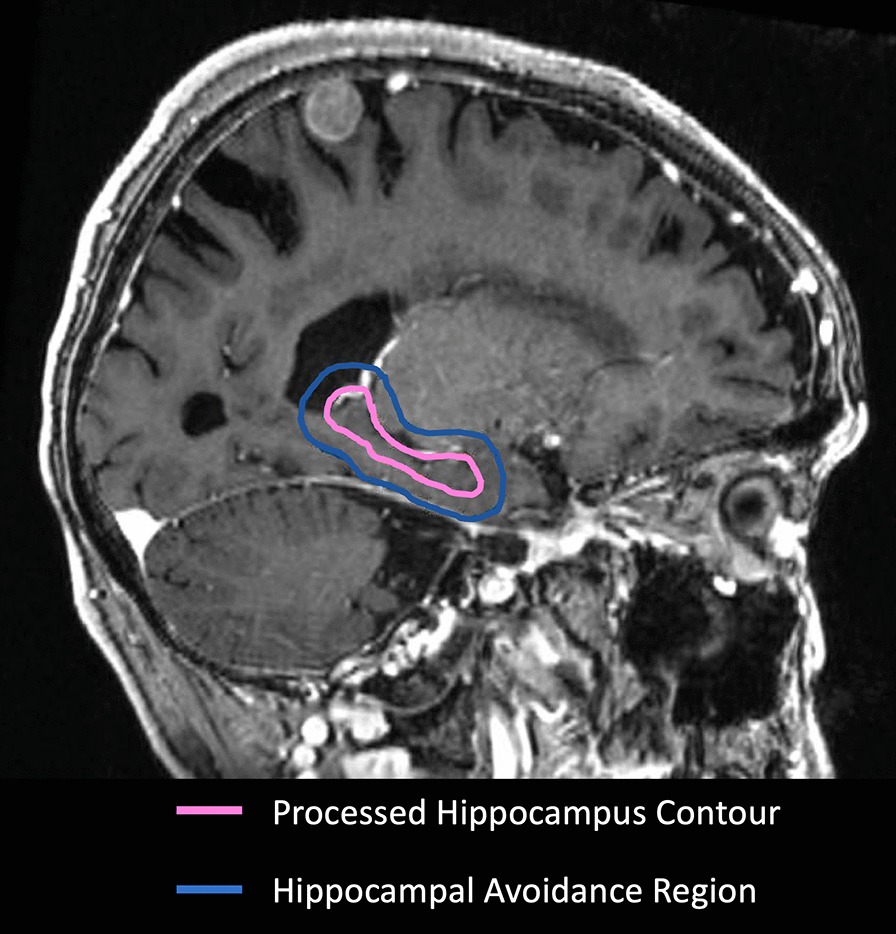
Representative case with processed hippocampus segmentation and hippocampal avoidance region

A summary of scan characteristics is presented in Table 1. The scans were split approximately evenly between 1.5 and 3.0 T systems. Sixteen (16%) cases had a resection cavity on the scan. Of the 100 cases, 99 (99%) had acceptable automated hippocampi segmentations without any manual intervention (Fig. 1). Time for review of hippocampi segmentations was not specifically recorded, but we estimate that it took less than 30 s per patient to review the contours and verify accuracy.

**Table 1:** Summary of MRI characteristics for all cases

		N (%)
Magnet strength (T)	1.5	53 (53%)
	3	47 (47%)
Slice thickness (mm)	1	94 (94%)
	1.2	1 (1%)
	1.5	1 (1%)
	2	4 (4%)
IV contrast	Yes	98 (98%)
	No	2 (2%)
Scanner model	GE SignaHDxt 1.5T	53 (53%)
	GE SignaHDxt 3.0T	28 (28%)
	GE Discovery MR750 3.0T	10 (10%)
	GE Discovery MR750w 3.0T	9 (9%)
Resection cavity	Yes	16 (16%)
	No	84 (84%)

Knowledge-based planning was applied to all cases, with individual optimization settings. Eleven (11%) cases were processed by an experienced dosimetrist. Eighty-nine (89%) cases were processed by a radiation oncologist after undergoing a 20-min training session with a dosimetrist. The median processing time for all plans was 9 min 59 s (range 6 min 53 s–13 min 31 s); variation in processing time depended primarily on how many users were using the server at time of plan generation. There was no difference in planning time between the dosimetrist and the radiation oncologist.

One case required minor manual editing at the junction of the hippocampus and the lateral ventricle due to the hippocampal segmentation extending 6 mm into the lateral ventricle. This case completed the pipeline using the automatically segmented contours, then underwent automated knowledge-based planning again using manually edited contours for PTV and PRV delineation. The change in volume between the automated and corrected PRVs was 3.87 cm^3^, less than 0.03% of the corrected PTV volume. Both plans met per-protocol dose-volume objectives for the corrected PTV and OARs.

**Fig. 2:**
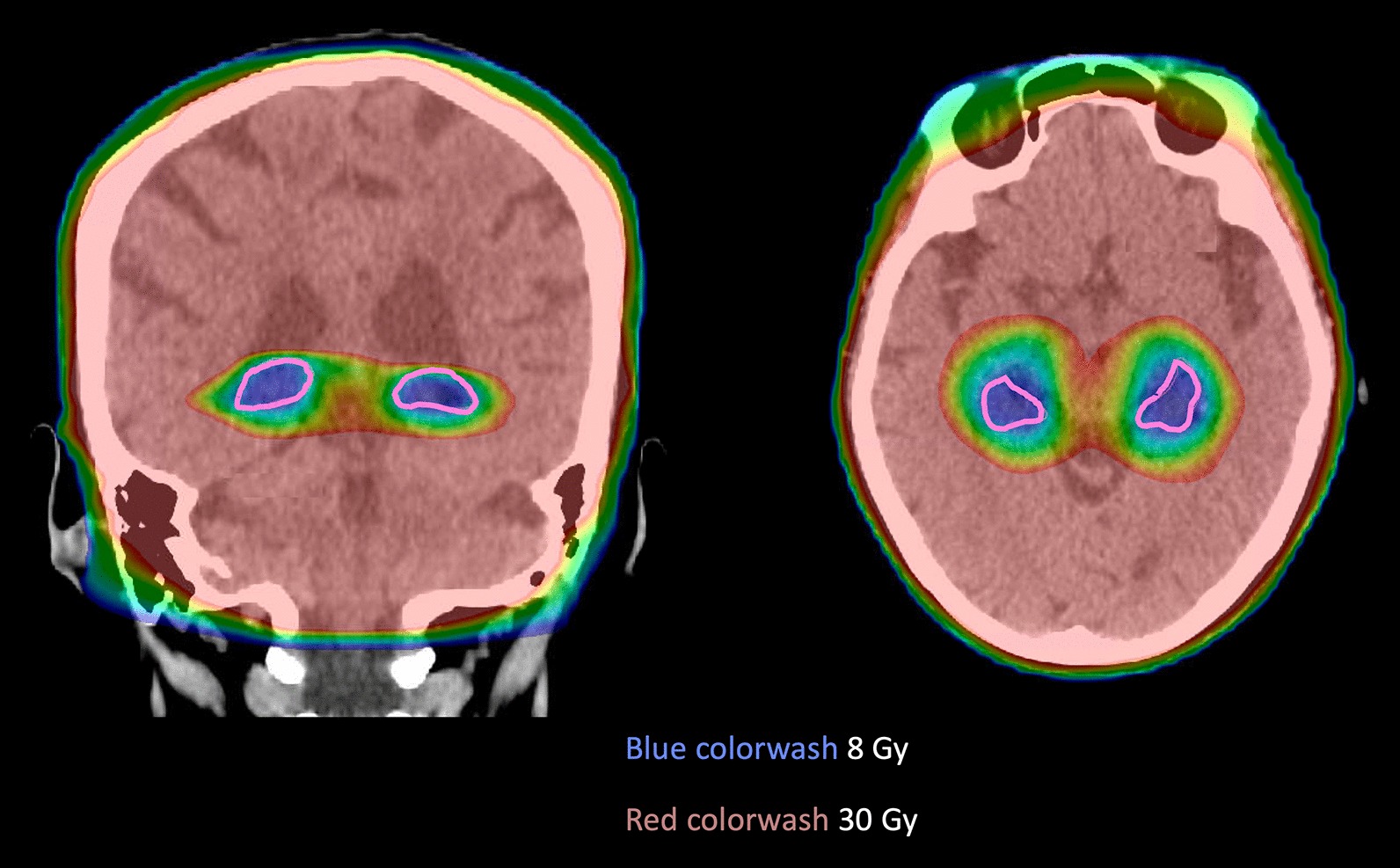
Representative case with dose in colorwash ranging from 8 to 30 Gy

All plans met acceptable dose-volume objectives for PTV and OARs per the NRG CC001 protocol (Table 2). A representative plan with dose overlay is shown in Fig. 2. PTV doses were per protocol for all plans, with D_2%_ below 37.5 Gy and D_98%_ greater than 25 Gy. For comparison, only 65.5% of plans on NRG CC001 met PTV goals per protocol, with 26.1% within acceptable variation [7]. In addition, 8.4% of plans on NRG CC001 were not scored or unable to be evaluated in terms of contouring and dose-volume analysis.

**Table 2.** Mean DVH metrics for HA-WBRT in current study compared to NRG CC001 constraints.

	NRG CC001		
	Per protocol	Acceptable variation	Current study Mean (range)
PTV D_2%_ (Gy)	< 37.5	37.5–40	34.3 (33.3–37.2)
PTV D_98%_ (Gy)	> 25	22.5–25	26.5 (25.3–28.8)
PTV V_30Gy_ (%)	> 95	90–95	95 (95–95)
Hippocampus D_100%_ (Gy)	< 9	9–10	8.1 (7.4–9.1)
Hippocampus D_max_ (Gy)	< 16	16–17	13.1 (9.7–15.7)
Optic Nerves D_max_ (Gy)	< 30	30–37.5	29.8 (25.4–32.3)
Optic Chiasm D_max_ (Gy)	< 30	30–37.5	30.1 (29.0–32.0)

*D* dose, *PTV* planning target volume

Dose to bilateral hippocampi was per protocol for 99 plans (99%), with one plan delivering D_100%_ of 9.05 Gy (per protocol was D_100%_ ≤ 9.0 Gy, with 9–10 Gy acceptable variation). Hippocampal D_max_ was less than the per protocol recommendation of 16 Gy for all plans. Forty-three plans (43%) met OAR constraints for optic structures, and the remaining 57 plans (57%) were within acceptable variation. The highest D_max_ for any optic structure across all plans was 32.3 Gy, well below the protocol maximum of 37.5 Gy. On NRG CC001, 42.5% of plans met OAR constraints per protocol, and 48.3% were within acceptable variation [7]. No plans in this study had unacceptable dose to OARs, compared to 0.8% of manually generated plans from NRG CC001.

## Discussion

Randomized clinical trial results have established HA-WBRT as superior to WBRT for preservation of neurocognitive function and quality of life [6, 7]. Using commercially available software for hippocampal contours and knowledge-based planning, we have established a workflow to generate automated HA-WBRT plans with meaningful efficiency. Standard clinical MRI data were used from either 1.5 or 3.0 T systems. Automated hippocampal volumes were accurate without any manual intervention in 99% of cases. Knowledge-based planning typically required approximately 10 min and yielded HA-WBRT plans that were more frequently adherent to the NRG CC001 protocol than the manually generated plans actually used in that trial [7].

Hippocampal-avoidant WBRT gained attention after publication of the results from RTOG 0933, and the recent results from NRG CC001 demonstrate improved patient outcomes over standard WBRT. Particularly as cancer therapies have improved survival rates, preservation of neurocognitive function and quality of life is increasingly important. However, manual hippocampal contouring can be challenging with high interobserver variability [16, 17], and inverse plan optimization can require multiple iterations to fulfill constraints with generation of helping structures. We have shown that automated tools could be integrated into this complex process to improve both efficiency and plan quality. While automation may streamline radiation planning, it is clearly imperative that all contours be verified before a plan is delivered. The OAR contours and plan review process was not timed in this study but is estimated to have taken on the order of a few minutes per case; this should be acknowledged as an essential component of the clinical routine for all radiation oncologists.

The sole automatically segmented hippocampal contour reported here as having an error only extended 6 mm into the lateral ventricle. The change in volume between the uncorrected and corrected PRVs (i.e., hippocampus plus uniform 5 mm margin) was 3.87 cm^3^, less than 0.03% of the corrected PTV volume. This small error in the uncorrected PRV had no meaningful impact on dose to the fairly distant other OARs (optic structures). The corrected PRV was within the uncorrected PRV and thus was spared with either plan. The minute difference in PTV volumes was also too small to significantly change any dose-volume measurements for target coverage. Given that 100% of plans based on automated contours were acceptable in this study, it appears reasonable for a radiation oncologist using this workflow to simply review the contours and plan at the same time. If automated hippocampal contours were meaningfully inaccurate for an outlier patient, a new plan could be quickly generated after manual correction of the contours.

The knowledge-based plans provided similar dose distributions to previously published manual and automated planning studies [8, 18–20]. In the studies from Gondi et al. and Nevelsky et al., plans were created using nine linac-based IMRT fields, while Krayenbuehl et al. used four non-coplanar arcs and Wang et al. studied both IMRT and VMAT approaches [8, 18–20]. Our plan metrics differ most notably in improved PTV coverage at the expense of slightly higher hot spots, compared to Krayenbuehl et al. [18], and decreased maximum dose to optic structures, in relation to the other studies [19, 20]. The automated workflow presented here also demonstrated a consistent plan quality across cases, with none exceeding protocol constraints. We did not perform manual checks during the knowledge-based planning process or prior to plan evaluation, and planning time primarily depended on server load.

One criticism of typical WBRT is poor local control of existing brain metastases after 30 Gy. A recent single-arm feasibility study investigated the utility of a simultaneous integrated boost (SIB) technique with WBRT [21]. The investigators prescribed 30 Gy in 12 fractions to the brain and simultaneously boosted metastases and resection cavities to 42 or 51 Gy. Comparison to propensity matched patients treated with conventional WBRT demonstrated increased intracranial progression-free survival and overall survival with HA-WBRT with SIB. The ongoing HIPPORAD trial (﻿NOA-14, ARO 2015–3, DRKS00004598) will further study this method. If that approach is successful, further development of the workflow presented here could incorporate SIB technique into knowledge-based planning.

Despite the recent publication of randomized clinical trial data indicating improved cognitive preservation and patient-reported quality of life with use of HA-WBRT over traditional WBRT, some centers may face challenges employing this technique. Compared to historical WBRT using two opposed lateral beams, HA-WBRT uses IMRT planning that is more labor-intensive; requires more time for quality assurance and treatment delivery; and is associated with increased cost [22, 23]. VMAT techniques can decrease treatment delivery time compared to fixed-field IMRT. Our study used 4 full arcs rather than 2–3 arcs used in some other studies. The publicly available knowledge-based planning routine was designed for a template using 4 full arcs, but could be further optimized in the future to use fewer arcs to minimize treatment delivery time [20, 24, 25]. Meanwhile, the automated workflow described here addresses some of the challenges of HA-WBRT adoption by decreasing physician and dosimetry burden.

There are some limitations to our study. The time for importation of automated segmentations and generation of standard OAR contours was not included in the planning time measurement. While an important component of the clinical routine, import of an MRI series would also be necessary for manual delineation of hippocampi and generally estimated to require 1–2 min per case. The hippocampal contours were generated automatically (except for minor manual edits in 1% of cases); although not timed here, these contours are typically generated in less than 8 min, per the software vendor [13, 14]. Standard OARs had been contoured previously and were also not timed. Future investigations could include automated contours of these standard structures, which are generally familiar and routinely included in most brain radiotherapy plans. The inclusion of automatically contoured standard OARs into this process would make the system completely autonomous, i.e. a class solution pipeline that takes as input standard imaging and outputs a complete HA-WBRT plan with no human-driven parameters.

## Conclusions

An automated pipeline harnessing the efficiency of commercially available artificial intelligence tools can consistently generate clinically acceptable VMAT HA-WBRT plans with minimal manual intervention. This process could improve clinical efficiency for a treatment established to improve patient outcomes over standard WBRT.

## Data Availability

The datasets used and analyzed during the current study are available from the corresponding author on reasonable request.

## Abbreviations

WBRT: Whole-brain radiotherapy
SRS: Stereotactic radiosurgery
HA-WBRT: Hippocampal-avoidant whole-brain radiotherapy
IMRT: Intensity-modulated radiotherapy
OARs: Organs-at-risk
PTV: Planning target volume
VMAT: Volumetric modulated arc therapy
DVH: Dose-volume histogram
SIB: Simultaneous integrated boost
